# Fingolimod potentiates minocycline against multidrug-resistant *Elizabethkingia*

**DOI:** 10.3389/fcimb.2026.1924132

**Published:** 2026-09-30

**Authors:** Jie Sun, Qiang Feng, Can Yang, Yulong Zong, Rongliang Gao, Li Shen, Fangyou Yu, Yongpeng Shang

**Affiliations:** 1 Department of Clinical Laboratory, The Affiliated Taian City Central Hospital of Qingdao University, Taian, China; 2 Department of Clinical Laboratory, Shanghai Pulmonary Hospital, School of Medicine, Tongji University, Shanghai, China

**Keywords:** drug repurposing, *Elizabethkingia* species, fingolimod, minocycline, synergy

## Abstract

**Objectives:**

*Elizabethkingia* spp. are intrinsically resistant to multiple antimicrobial classes and are emerging nosocomial pathogens. This study evaluated the antibacterial activity of fingolimod against *Elizabethkingia* and its interaction with minocycline.

**Methods:**

Sixteen residual clinical isolates were examined. Baseline single-agent susceptibility was assessed by a single disk-diffusion determination and by VITEK 2. Fingolimod MIC and MBC values were measured by broth microdilution. Biofilm formation was quantified by crystal violet staining. Fingolimod–minocycline interactions were evaluated by checkerboard and time-kill assays. Preliminary protective activity was examined in a *Galleria mellonella* model infected with EA12.

**Results:**

Disk diffusion and VITEK 2 showed method-dependent discordance for piperacillin–tazobactam. Fifteen of 16 isolates formed biofilm; 13 of these 15 were weak and two were moderate producers. Fingolimod MICs ranged from 6.25 to 25 μg/mL. Fingolimod reduced the minocycline MIC by 4–8-fold in six synergistic isolates. The combination produced preliminary, isolate-specific protection in *Galleria mellonella* infected with EA12.

**Conclusion:**

Fingolimod exhibits antibacterial activity against *Elizabethkingia* spp. and potentiates minocycline in a subset of isolates. These findings support further development of fingolimod-based combination strategies and optimization of the fingolimod scaffold.

## 1 Introduction

*Elizabethkingia* spp. are emerging multidrug-resistant Gram-negative pathogens. They cause severe infections, particularly in immunocompromised patients and neonates (Anil et al., 2023; Lau et al., 2016). *Elizabethkingia anophelis* (EA) is the predominant species in many clinical series (Chew et al., 2018; Nicholson et al., 2018). The genus is intrinsically resistant to multiple antimicrobial classes. Chromosomal *blaB*, *blaGOB*, and *blaCME* contribute to resistance to most β-lactams, including carbapenems (Lau et al., 2016; Lin et al., 2019). Minocycline often retains *in vitro* activity, whereas susceptibility to levofloxacin, trimethoprim–sulfamethoxazole, and rifampicin is variable (Chiu et al., 2021; Han et al., 2017; Lin et al., 2018).

Drug repurposing can reveal antibacterial activities outside the original therapeutic indication (Di Bonaventura et al., 2023; Gilbert-Girard et al., 2020). Fingolimod is an S1P receptor modulator approved for relapsing multiple sclerosis. Previous studies reported antibacterial or antibiofilm activity in several bacterial species (Geng et al., 2024; Gilbert-Girard et al., 2020; Shang et al., 2025). These observations provide a rationale for testing the fingolimod scaffold against intrinsically resistant Gram-negative pathogens.

The antibacterial mechanism of fingolimod in *Elizabethkingia* remains unknown. We therefore evaluated its *in vitro* activity against clinical isolates and tested its interactions with five antibiotics. Minocycline was examined across the full isolate set and in a preliminary *Galleria mellonella* infection model.

## 2 Materials and methods

### 2.1 Bacterial strains, culture conditions, and antimicrobial compounds

Sixteen residual *Elizabethkingia* isolates remaining after routine diagnostic procedures were used. No patient identifiers or isolate–patient linkage data were available. Isolates were stored in cryopreservation medium containing 20% glycerol at −80 °C. Strains were cultured at 37 °C in tryptic soy broth (TSB; OXOID, Basingstoke, UK) and on Mueller–Hinton (MH) agar (OXOID) or Columbia blood agar (Komajia Culture Medium, Shanghai, China). Levofloxacin (CAS 100986-85-4), tetracycline (CAS 60-54-8), and vancomycin (CAS 1404-90-6) were purchased from MedChemExpress. Minocycline hydrochloride (CAS 13614-98-7) and fingolimod hydrochloride (CAS 162359-56-0) were obtained from TargetMol. Rifampicin (CAS 13292-46-1) was purchased from Sangon Biotech. Piperacillin sodium (MCE, HY-B1286; CAS 59703-84-3) and tazobactam sodium (MCE, HY-W009168; CAS 89785-84-2) were used for broth microdilution confirmation. Tazobactam was fixed at 4 μg/mL. Stock solutions were aliquoted and stored at −20 °C.

### 2.2 Species identification and antimicrobial susceptibility testing

VITEK MS (bioMérieux, France) was used for preliminary identification. Because this platform could not reliably resolve the isolates at the species level, *Elizabethkingia* spp. assignments were confirmed by whole-genome sequencing performed by our collaborators. The sequence data have been deposited and published (Shang et al., 2026). Routine antimicrobial susceptibility testing (AST) results were obtained with VITEK 2 AST-N335 cards. *Pseudomonas aeruginosa* ATCC 27853 was used for quality control (QC) of routine AST.

Multidrug resistance was operationally defined as non-susceptibility to at least one agent in three or more antimicrobial categories based on VITEK 2 results. Disk diffusion was performed once for each isolate as an initial descriptive single-agent screen. A 0.5 McFarland suspension was inoculated onto MH agar, and disks containing 14 antibiotics (OXOID) were applied. Plates were incubated at 37 °C for 20 h. Zone diameters were interpreted using the adapted criteria specified in **Supplementary Table 1**. Disk results were not treated as definitive evidence of susceptibility.

### 2.3 Biofilm formation assay

Biofilm formation was quantified by crystal violet staining. Each isolate was diluted 1:100 in TSB and grown to logarithmic phase. Cultures were transferred to 96-well polystyrene plates containing TSB with 0.5% glucose (TSBG) and incubated statically at 37 °C for 24 h. Wells were washed three times with 200 μL PBS, fixed with 200 μL methanol for 15 min, stained with 1% crystal violet for 30 min, and washed. Bound stain was dissolved in 33% acetic acid, and OD_600_ was measured. Each plate contained three sterile TSBG wells without bacterial inoculum as negative controls. No separate positive-control strain was included. ODc was calculated as the mean OD_600_ of the three negative-control wells plus three standard deviations. Isolates were classified as non-producers (OD ≤ ODc), weak (ODc < OD ≤ 2 × ODc), moderate (2 × ODc < OD ≤ 4 × ODc), or strong producers (OD > 4 × ODc) (Stepanovic et al., 2000). No drug or DMSO was present in this baseline biofilm assay.

### 2.4 MIC and MBC determination

Fingolimod MICs were determined by broth microdilution in cation-adjusted Mueller–Hinton broth (CAMHB; HaiBo, Qingdao, China). Log-phase cultures were adjusted to 0.5 McFarland (approximately 1.5 × 10^8^ CFU/mL) and diluted 100-fold in CAMHB. Each well contained 100 μL bacterial suspension and 100 μL drug-containing CAMHB, giving a final inoculum of approximately 7.5 × 10^5^ CFU/mL in 200 μL. Plates were incubated at 37 °C for 16–20 h. Minimum inhibitory concentrations (MIC) was the lowest concentration that prevented visible growth. Fingolimod was prepared as a 10-mg/mL stock in DMSO and diluted in CAMHB. The final DMSO concentration was ≤0.5% at the MIC concentrations tested. *Staphylococcus aureus* ATCC 29213 was included as a reference strain for fingolimod activity, not as a formal QC strain because no Clinical and Laboratory Standards Institute (CLSI) and the European Committee on Antimicrobial Susceptibility Testing (EUCAST) fingolimod QC range exists. For minimum bactericidal concentration (MBC) determination, 100 μL from wells without visible growth was plated on drug-free agar and incubated for 18–24 h. MBC was the lowest concentration producing a ≥99.9% reduction in viable counts.

### 2.5 Fractional inhibitory concentration

Drug interactions were evaluated by checkerboard broth microdilution. Fingolimod and antibiotic stock solutions were prepared at 10 mg/mL, and working solutions were diluted in CAMHB. Each 200-μL well contained 50 μL fingolimod solution, 50 μL antibiotic solution, and 100 μL bacterial suspension. A 0.5 McFarland suspension was diluted 100-fold before addition, producing a final inoculum of approximately 7.5 × 10^5^ CFU/mL. Plates were incubated at 37 °C for 20–24 h. Fractional inhibitory concentration index (FICI) was calculated as (MIC of drug A in combination/MIC of drug A alone) + (MIC of fingolimod in combination/MIC of fingolimod alone). Outcomes were defined as synergy (FICI ≤ 0.5), additivity (0.5 < FICI ≤ 1.0), indifference (1.0 < FICI < 4.0), or antagonism (FICI ≥ 4.0) (Cetin et al., 2013).

### 2.6 Time-kill assay

Time-kill assays were performed for three checkerboard-synergistic isolates (EA7, EA12, and EA14) and one additive isolate (EM8). Tested concentrations were selected from the corresponding checkerboard MIC results. Cultures were exposed to fingolimod, minocycline, their combination, or drug-free CAMHB. At 0, 4, 12, and 24 h, a 1 mL aliquot was collected, centrifuged, washed with sterile PBS to remove residual antimicrobial agents, and resuspended in 1 mL PBS. Undiluted samples and serial tenfold dilutions were plated at 100 μL on drug-free MH agar. Colonies were counted after incubation at 37 °C for 24 h. The lower limit of detection (LOD) was 10 CFU/mL (1 log_10_ CFU/mL). Values below this limit were reported as below the detection limit. Synergy was defined as a ≥2 log_10_ CFU/mL reduction for the combination relative to the most active single agent. Antagonism was defined as a ≥1 log_10_ CFU/mL increase for the combination compared with the most active single agent (Olsson et al., 2024).

### 2.7 *Galleria mellonella* infection models

*Galleria mellonella* larvae (approximately 250 mg) purchased from Tianjin Huiyude Biotechnology Co., Ltd. (Tianjin, China) were used to evaluate preliminary protective activity (Cutuli et al., 2019; Tsai et al., 2016). Larvae of uniform body weight were randomly allocated to experimental groups, and individuals with apparent melanization or physical injury were excluded prior to injection. EA12 was selected because it showed synergy in the checkerboard assay. Larvae were infected with approximately 1 × 10^6^ CFU. Each group contained eight larvae. At 30 min after infection, larvae received minocycline (5 mg/kg), fingolimod (2.5 mg/kg), or the combination in a 15-μL injection. The fingolimod-containing formulations contained 0.417% (v/v) DMSO. Survival was recorded every 12 h for 72 h. Additional uninfected groups received fingolimod, minocycline, or the combination to assess treatment-related toxicity. All experiments were performed in duplicate.

### 2.8 Statistical analysis

Analyses were performed in GraphPad Prism 10. Continuous data were analyzed using Student’s t-test or one-way ANOVA, as appropriate. Survival curves were analyzed by the Kaplan–Meier method and compared using the log-rank (Mantel–Cox) test. Bonferroni correction was applied to adjust *P* values for multiple comparisons. A corrected *P* value < 0.05 was considered statistically significant.

## 3 Results

### 3.1 Isolate collection and species identification

Sixteen residual isolates were available for analysis. Specimen types are reported in **Supplementary Table 1**. Whole-genome sequencing confirmed 15 isolates as EA and one as EM (Shang et al., 2026). No patient-level clinical information or isolate–patient linkage data were available.

### 3.2 Comparison of antimicrobial susceptibility testing methods

Baseline AST was assessed by a single disk-diffusion determination and by VITEK 2 (**Supplementary Table 1**). Categorical agreement was 100% for ceftazidime, imipenem, tobramycin, and levofloxacin. Agreement was 81.25% for cefepime and 68.75% for ciprofloxacin.

Piperacillin–tazobactam showed complete method-dependent discordance. In the single disk-diffusion determination, all 16 isolates were categorized as susceptible under the adapted criteria, whereas VITEK 2 categorized all isolates as resistant. Broth microdilution with tazobactam fixed at 4 μg/mL yielded piperacillin MICs of 4-16 μg/mL. These values were interpreted as susceptible according to the non-species-specific criteria applied in this study. They do not establish clinical susceptibility because *Elizabethkingia*-specific breakpoints are unavailable.

VITEK 2 categorized all isolates as resistant to ceftazidime, imipenem, and tobramycin, supporting the observed multidrug-resistant phenotype. Minocycline showed the highest activity, with all isolates categorized as susceptible. Doxycycline and trimethoprim–sulfamethoxazole susceptibility rates were 75% and 81.25%, respectively.

Interpretive criteria were unavailable for several agent–organism combinations. These results are therefore reported descriptively. The observed discordance underscores the need for *Elizabethkingia*-specific testing standards.

### 3.3 Biofilm formation

Fifteen of 16 isolates demonstrated measurable biofilm formation. Among these 15 isolates, 13 were weak producers and 2 (EA11 and EA12) were moderate producers. No isolate was a strong producer (**Figure 1**). The biofilm phenotype was not used to infer the mechanism of the fingolimod–minocycline interaction.

**Figure 1:**
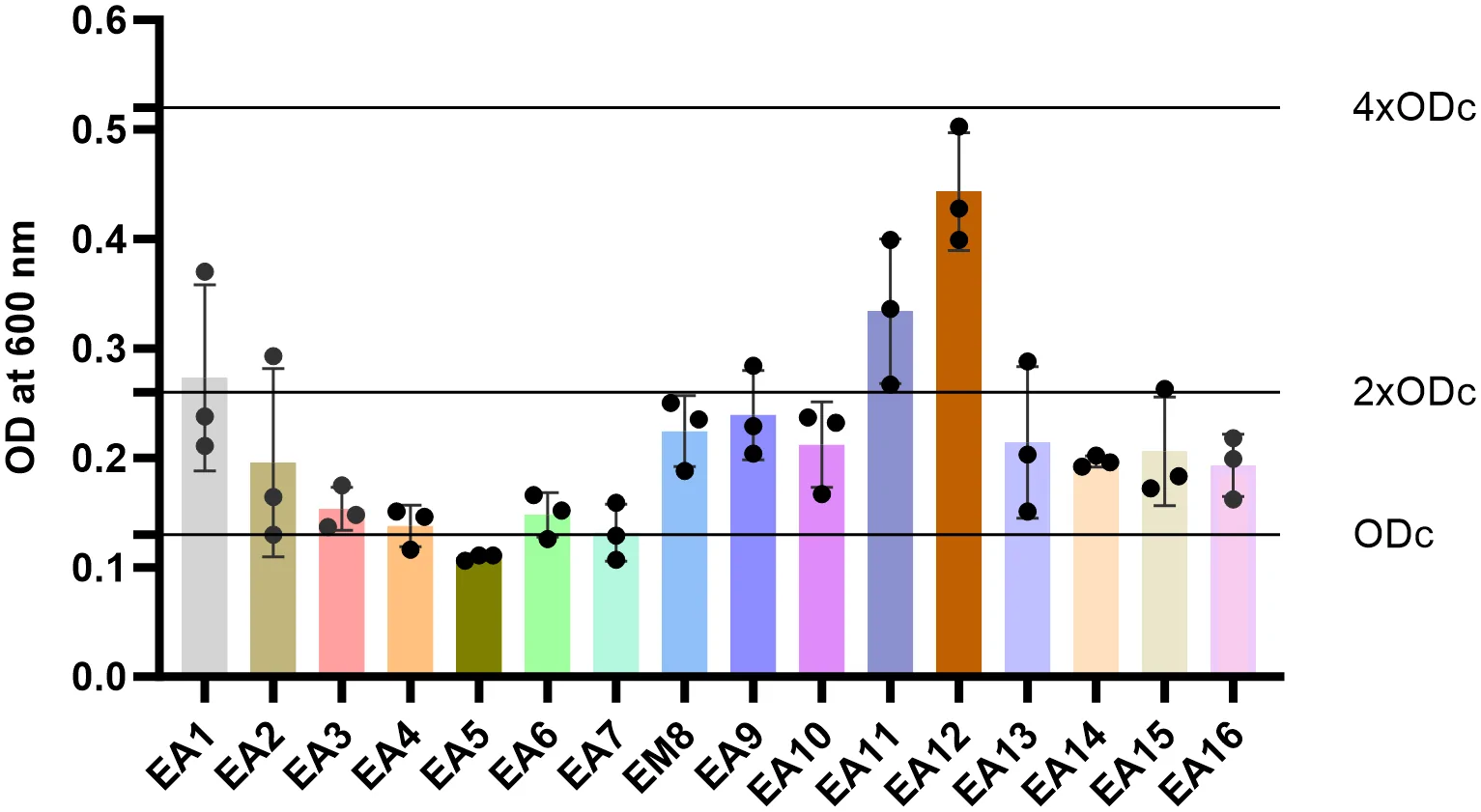
Biofilm formation by 16 isolates. Non-producer, OD ≤ ODc; weak, ODc < OD ≤ 2 × ODc; moderate, 2 × ODc < OD ≤ 4 × ODc; strong, OD > 4 × ODc. Bars show mean OD_600_; error bars show SD. Individual data points from three independent experiments are overlaid.

### 3.4 Fingolimod exhibits antibacterial activity against *Elizabethkingia* spp. *in vitro*

Fingolimod MICs ranged from 6.25 to 25 μg/mL. 15 of 16 isolates had MICs of 12.5–25 μg/mL. MBC values were ≥200 μg/mL, and MBC/MIC ratios were >8 for most isolates (**Table 1**). Thus, fingolimod showed consistent but modest, predominantly bacteriostatic activity under the tested conditions.

**Table 1:** Fingolimod MIC and MBC values for 16 *Elizabethkingia* isolates.

Isolate	MIC(μg/mL)	MBC (μg/mL)	MBC/MIC
EA1	25	>200	>8
EA2	25	>200	>8
EA3	12.5	>200	>16
EA4	12.5	200	16
EA5	12.5	200	16
EA6	25	>200	>8
EA7	25	>200	>8
EM8	12.5	>200	>16
EA9	12.5	>200	>16
EA10	12.5	>200	>16
EA11	12.5	200	16
EA12	25	>200	>8
EA13	6.25	200	32
EA14	12.5	200	16
EA15	25	>200	>8
EA16	25	>200	>8

EA13 had the lowest fingolimod MIC (6.25 μg/mL). Seven isolates had MICs of 25 μg/mL. These results motivated evaluation of fingolimod in combination with conventional antibiotics.

### 3.5 Fingolimod potentiates minocycline in a subset of isolates

Disk diffusion was used only for baseline single-agent AST. All combination screening was performed by checkerboard broth microdilution. Four isolates were initially tested with fingolimod combined with vancomycin, tetracycline, rifampicin, levofloxacin, or minocycline. The non-minocycline combinations produced additive or indifferent interactions (FICI 0.625–1.25). No antagonism was observed (**Supplementary Figure 1**).

The fingolimod–minocycline combination was then tested against all 16 isolates. Six isolates (37.5%; EA1, EA6, EA7, EA12, EA14, and EA16) showed synergy. The remaining ten isolates showed additivity (**Figure 2**; **Table 2**). Fingolimod reduced the minocycline MIC by 4–8-fold in synergistic isolates. The combination also reduced the fingolimod concentration required for activity.

**Figure 2:**
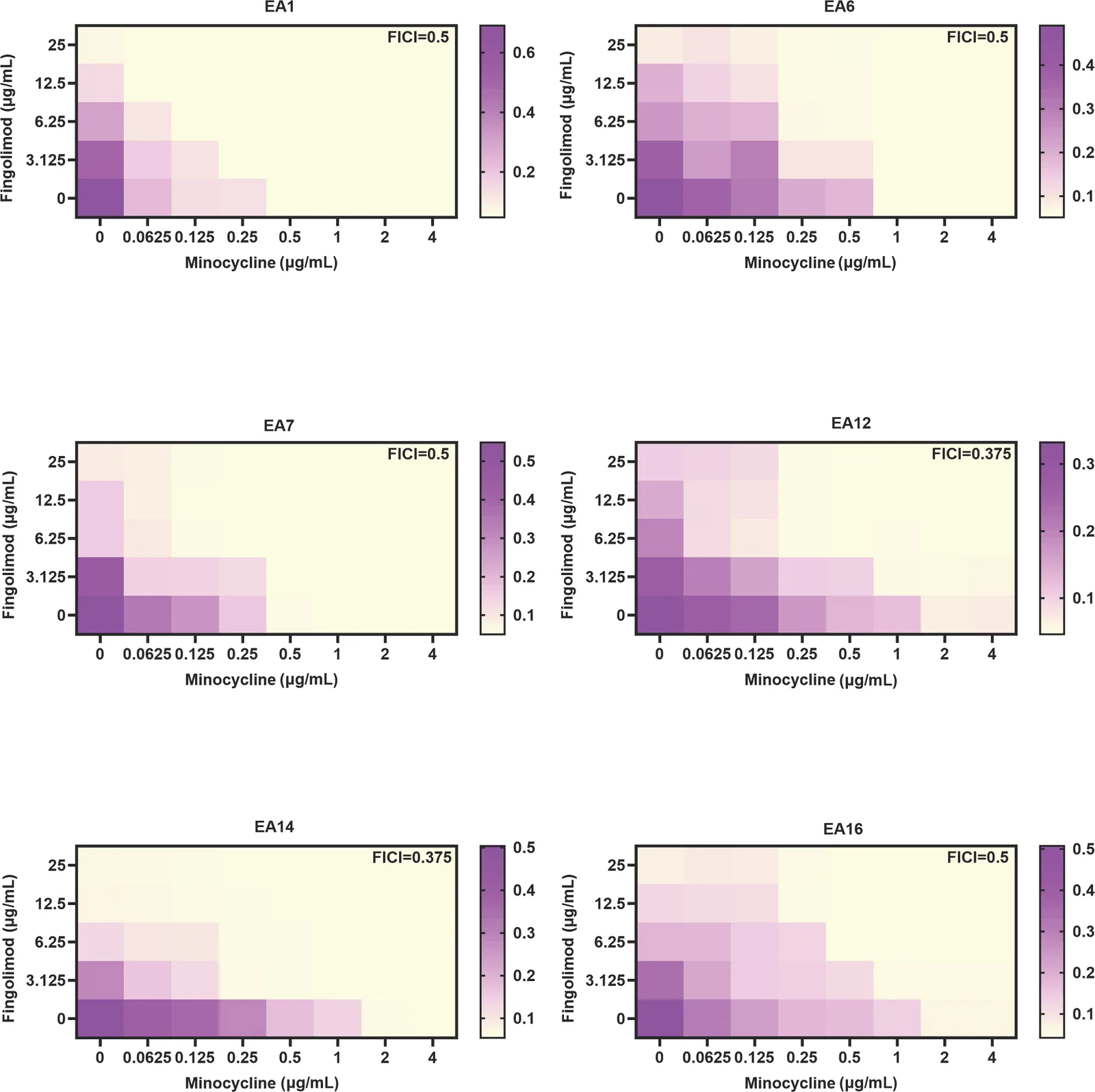
Checkerboard results for the six isolates showing fingolimod–minocycline synergy. FICI ≤ 0.5 indicates synergy.

**Table 2:** Checkerboard interaction between fingolimod and minocycline for 16 isolates.

Isolate	Individual MIC(μg/mL)		Combined MIC(μg/mL)		FICI	Interpretation
	Fingolimod	Minocycline	Fingolimod	Minocycline		
EA1	25	0.5	6.25	0.125	0.5	Synergy
EA2	25	0.5	3.125	0.25	0.625	Additivity
EA3	12.5	0.25	3.125	0.125	0.75	Additivity
EA4	12.5	0.25	6.25	0.0625	0.75	Additivity
EA5	12.5	0.5	3.125	0.25	0.75	Additivity
EA6	25	1	6.25	0.25	0.5	Synergy
EA7	25	0.5	6.25	0.125	0.5	Synergy
EM8	12.5	0.5	3.125	0.25	0.75	Additivity
EA9	12.5	0.5	3.125	0.25	0.75	Additivity
EA10	12.5	0.5	6.25	0.25	1	Additivity
EA11	12.5	0.5	6.25	0.125	0.75	Additivity
EA12	25	2	6.25	0.25	0.375	Synergy
EA13	6.25	0.5	3.125	0.125	0.75	Additivity
EA14	12.5	2	3.125	0.25	0.375	Synergy
EA15	25	1	3.125	0.5	0.625	Additivity
EA16	25	2	6.25	0.5	0.5	Synergy

### 3.6 Time-kill assays validate selected checkerboard interactions

Time-kill assays were limited to three checkerboard-synergistic isolates and one additive isolate. This selected subset does not represent the full isolate collection.

For EA7 and EA12, fingolimod alone produced an early reduction at 4 h, followed by regrowth at 12 h. The combination reduced viable counts below the detection limit by 4 h and maintained this effect through 24 h. For EA14, the combination produced a sustained >2 log_10_ CFU/mL reduction by 12 h. For additive isolate EM8, the combination did not improve activity beyond the more active monotherapy (**Figure 3**).

**Figure 3:**
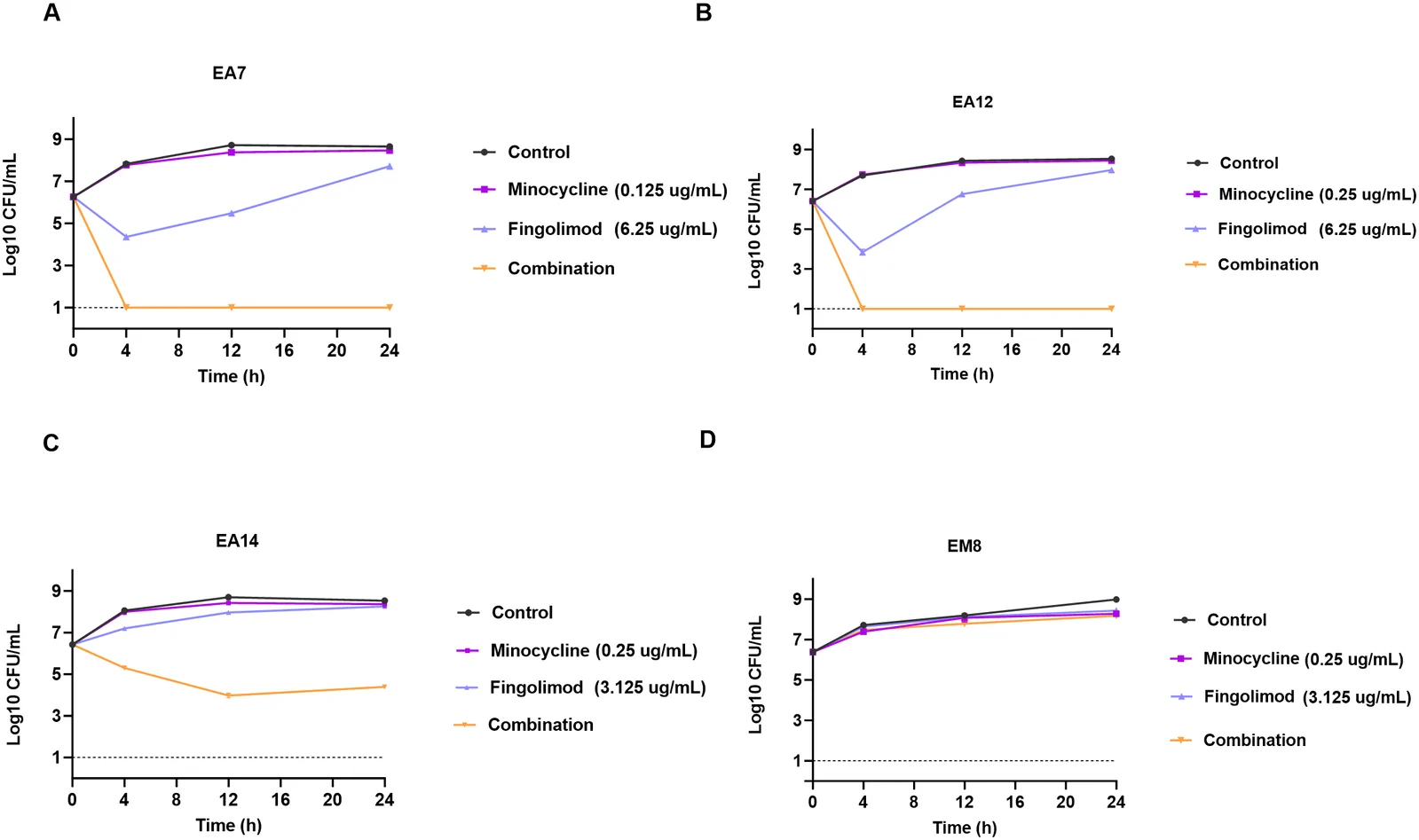
Time-kill results for three synergistic isolates (EA7, EA12, and EA14) and one additive isolate (EM8). Concentrations were selected from checkerboard MIC results. The detection limit was 10 CFU/mL (1 log_10_ CFU/mL); values below this limit are indicated as below the detection limit.

These results functionally validated the checkerboard interaction in the selected synergistic isolates. They do not establish uniform activity across *Elizabethkingia* spp.

### 3.7 Preliminary protective activity in the *Galleria mellonella* infection model

EA12 was selected as a checkerboard-synergistic isolate. The combination produced partial protection relative to either monotherapy, with approximately 25% survival at 72 h (**Figure 4**). Uninfected larvae receiving fingolimod, minocycline, or the combination showed no reduction in survival under the tested conditions (**Supplementary Figure 2**). Kaplan–Meier survival analysis revealed significant overall differences in larval survival across the five experimental groups (*P* < 0.0001, log-rank (Mantel–Cox) test). Pre-specified pairwise comparisons were performed with Bonferroni correction. Combination therapy significantly improved survival compared with the untreated infection group (raw *P* = 0.011, Bonferroni-adjusted *P* = 0.044 <0.05). In contrast, survival did not differ significantly between the untreated group and larvae receiving fingolimod or minocycline monotherapy. While our findings are isolate-specific to EA12, they demonstrate preliminary protective activity and provide *in vivo* proof of concept for the synergistic interaction of this combination.

**Figure 4:**
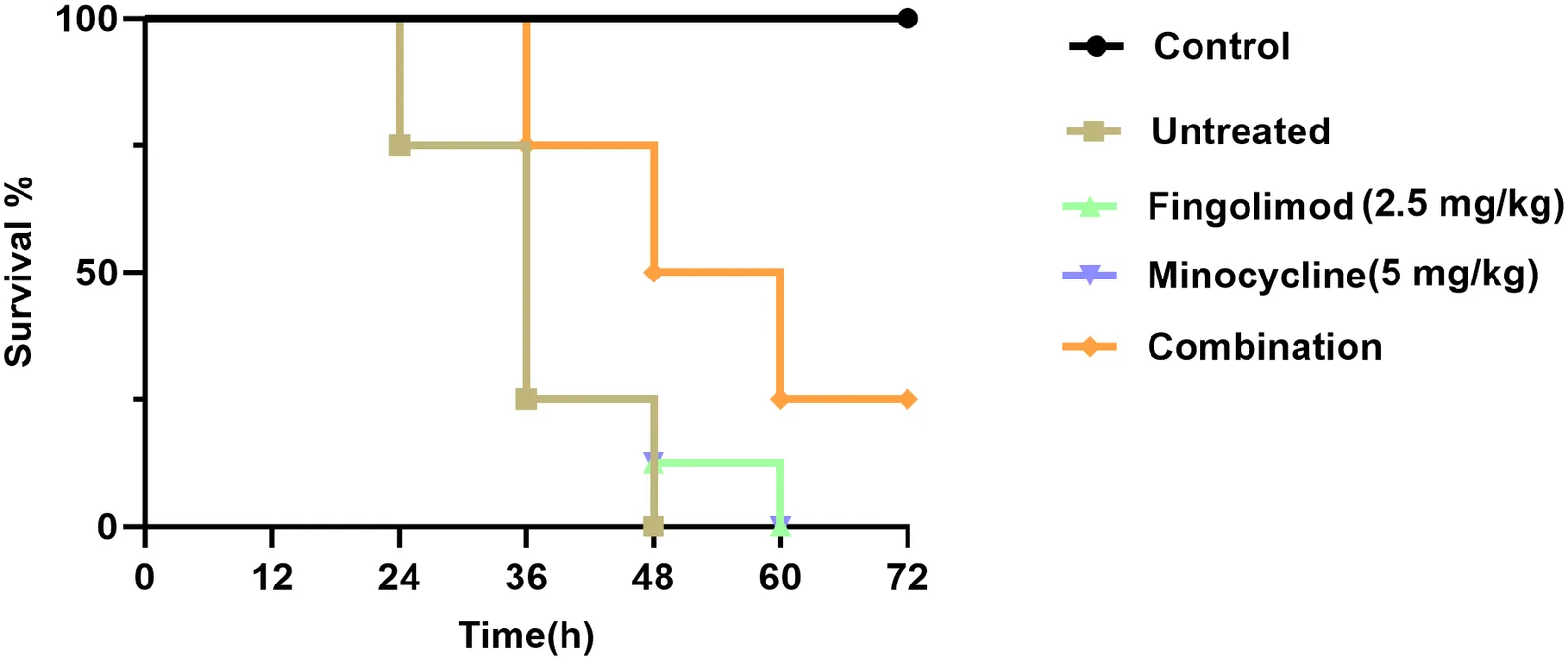
Survival of *Galleria mellonella* infected with EA12 and treated with PBS (untreated), minocycline (5 mg/kg), fingolimod (2.5 mg/kg), or their combination (n = 8 per group). Uninfected larvae served as the control group. The global difference among the five groups was assessed by the log-rank (Mantel–Cox) test. Four *a priori* pre-specified pairwise log-rank comparisons were performed using the untreated infection group as the reference (versus control, fingolimod, minocycline, and combination), with Bonferroni correction applied. All 10 pairwise raw *P* values and the four adjusted *P* values are summarized in **Supplementary Table 2**.

## 4 Discussion

*Elizabethkingia* spp. display intrinsic resistance to multiple antimicrobial classes. In this study, VITEK 2 categorized every isolate as resistant to ceftazidime, imipenem, and tobramycin. This pattern supports the multidrug-resistant phenotype observed in the isolate set. Minocycline retained the most consistent *in vitro* activity.

AST interpretation remains difficult because *Elizabethkingia*-specific breakpoints are unavailable. Disk diffusion and VITEK 2 were concordant for several agents but diverged for ciprofloxacin, cefepime, and piperacillin–tazobactam. For piperacillin–tazobactam, disk diffusion, VITEK 2, and broth microdilution produced different categorical implications. We therefore describe this result as method-dependent discordance rather than platform misinterpretation.

Fingolimod showed modest, predominantly bacteriostatic activity against *Elizabethkingia* spp. Its interaction with minocycline was more informative. Six isolates showed checkerboard synergy, with a 4–8-fold reduction in the minocycline MIC. Selected synergistic isolates also showed enhanced killing in time-kill experiments. The antibacterial mechanism in *Elizabethkingia* remains unresolved.

The translational interpretation must consider human exposure. Fingolimod is >99.7% protein bound and extensively distributed into tissues (David et al., 2012) (FDA GILENYA prescribing information). At the approved dose, systemic exposure, particularly the unbound fraction, is below the concentrations required for direct antibacterial activity. The present data therefore do not support immediate systemic use of fingolimod for *Elizabethkingia* infection. Structural optimization has generated fingolimod derivatives with improved antibacterial potency (Zore et al., 2021). The translational value lies in combination strategies and optimization of the fingolimod scaffold.

The *Galleria mellonella* experiment provides only preliminary evidence. It used one synergistic isolate, EA12, and produced partial protection. The model also cannot assess human adaptive immunity or fingolimod-induced lymphocyte sequestration. Fingolimod is an S1P receptor modulator, and mammalian studies must evaluate immune effects, PK/PD, toxicity, and drug-drug interactions (Brinkmann et al., 2010; Chun et al., 2019).

This study has several limitations. The isolate set was small and contained predominantly EA. Combination screening was limited, and time-kill validation used a selected subset. Disk diffusion was performed once per isolate as a descriptive screen. The *Galleria mellonella* experiment used a single isolate. Further work should define the mechanism of interaction, evaluate optimized derivatives, and test exposure–response relationships in mammalian infection models.

## Data Availability

The original contributions presented in the study are included in the article/**Supplementary Material**, further inquiries can be directed to the corresponding author/s.
