# Supplementary material for: Fingolimod potentiates minocycline against multidrug-resistant Elizabethkingia
Source: Front Cell Infect Microbiol. 2026 Sep 30;16:1924132. doi: 10.3389/fcimb.2026.1924132 (PMC13661465; doi:10.3389/fcimb.2026.1924132)
Supplement: Supplementary file 1 [file Table1.docx]

Supplementary Figure 1. (A) The checkerboard experiment of fingolimod and vancomycin(n=4); (B) The checkerboard experiment of fingolimod and tetracycline(n=4); (C) The checkerboard experiment of fingolimod and levofloxacin(n=4); (D) The checkerboard experiment of fingolimod and rifampin(n=4). FICI values are shown in the top right of each checkerboard, with interpretation as follows: synergy, ≤0.5; additive, 0.5-1.0, indifference,1.0-4.0; and antagonism≥4.

Supplementary Figure 2. Toxicity evaluation of fingolimod, minocycline, or their combination in uninfected *Galleria mellonella* larvae (control: DMSO-only treatment; n = 8 per group).
