# Supplementary material for: Fingolimod potentiates minocycline against multidrug-resistant Elizabethkingia
Source: Front Cell Infect Microbiol. 2026 Sep 30;16:1924132. doi: 10.3389/fcimb.2026.1924132 (PMC13661465; doi:10.3389/fcimb.2026.1924132)
Supplement: Supplementary file 2 [file Table3.docx]

Supplementary Table 2. Pairwise log-rank (Mantel-Cox) comparisons of *Galleria mellonella* larval survival among experimental groups.

| Comparison | *χ2* | *df* | Raw *P* value | Bonferroni-adjusted *P* value |
| --- | --- | --- | --- | --- |
| Control vs Untreated | 15.864 | 1 | <0.001 | <0.004 |
| Control vs Fingolimod | 16.732 | 1 | <0.001 | Not pre-specified;no adjustment applied |
| Control vs Minocycline | 16.732 | 1 | <0.001 | Not pre-specified; no adjustment applied |
| Control vs Combination | 9.135 | 1 | 0.003 | Not pre-specified; no adjustment applied |
| Untreated vs Fingolimod | 0.141 | 1 | 0.708 | 1.000 |
| Untreated vs Minocycline | 0.141 | 1 | 0.708 | 1.000 |
| Untreated vs Combination | 6.533 | 1 | 0.011 | 0.044 |
| Fingolimod vs Minocycline | 0.000 | 1 | 1.000 | Not pre-specified; no adjustment applied |
| Fingolimod vs Combination | 4.709 | 1 | 0.030 | Not pre-specified; no adjustment applied |
| Minocycline vs Combination | 4.709 | 1 | 0.030 | Not pre-specified; no adjustment applied |

Footnote: The overall intergroup difference was assessed using the log-rank test (*χ²*=28.686, *df*=4, *P*<0.0001). All pairwise log-rank comparisons are listed with unadjusted raw *P* values. Bonferroni correction was applied only to the four pre-specified comparisons comparing the untreated infection group against the remaining experimental groups; adjusted *P* values for these four comparisons are shown. No correction was performed for other pairwise comparisons.
